# Lower Free T3 Levels Linked to Poorer Outcomes in Chronic Obstructive Pulmonary Disease Patients with Acute Hypercapnic Respiratory Failure

**DOI:** 10.2478/jccm-2024-0002

**Published:** 2024-01-30

**Authors:** Türkay Akbaş, Harun Güneş

**Affiliations:** Düzce University, School of Medicine, Düzce, Türkiye; Balıkesir University, School of Medicine, Balıkesir, Türkiye

**Keywords:** hypercapnic respiratory failure, critical illness, free triiodothyronine, mortality predictor, non-thyroidal illness syndrome

## Abstract

**Aim of the Study:**

Non-thyroidal illness syndrome (NTIS) is often observed in critically ill patients. This study aimed to examine thyroid hormone changes in patients with chronic obstructive pulmonary disease (COPD) experiencing acute hypercapnic respiratory failure (AHRF) and to evaluate the impact of these alterations on clinical outcomes.

**Materials and Methods:**

This retrospective investigation involved 80 COPD patients (age 71.5±9.5 years; 57.5% male) admitted to the intensive care unit (ICU) due to AHRF. NTIS was identified when free triiodothyronine (fT3) levels were below the lower limit, and thyroid-stimulating hormone (TSH) and free thyroxine (fT4) levels were within the normal range or below the lower limits.

**Results:**

NTIS was detected in 63.7% of the patients. Decreased fT3 levels were found in 36.3% of the patients, reduced T4 levels in 33.8%, and diminished TSH levels in 15%. Patients with low fT3 levels exhibited elevated C-reactive protein levels, white blood cell counts, and APACHE II scores, necessitated vasopressor infusion more frequently during their ICU stay, and had increased mortality. The in-hospital mortality rate was 28.8%. Logistic regression analysis revealed that fT3 level (odds ratio [OR]., 0.271; 95% confidence interval [CI]., 0.085–0.865; p=0.027), APACHE II score (OR, 1.155; 95% CI, 1.041–1.282; p=0.007), and vasopressor use (OR, 5.426; 95% CI, 1.439–20.468; p=0.013) were crucial predictors of in-hospital mortality.

**Conclusions:**

A high prevalence of NTIS is observed in COPD patients with AHRF, with low fT3 levels frequently observed. The presence of lower levels of fT3 is associated with a greater severity of the disease and a significant prognostic indicator.

## Introduction

Chronic obstructive pulmonary disease (COPD) is a progressive disorder with irreversible airflow obstruction. The primary cause of COPD is a chronic inflammation of the lungs due to harmful particles and gases, predominantly from cigarette smoke [1]. Acute respiratory failure disrupts the clinical course of COPD and accounts for approximately 2% of all ICU admissions [2]. COPD is also a systemic disease, leading to various organ dysfunctions, such as muscle loss, depression, osteoporosis, cardiovascular diseases, and neuroendocrine changes [1]. Systemic inflammation originating from the lungs, whether during stable disease or acute exacerbations, contributes to developing COPD comorbidities [3].

Numerous studies have investigated thyroid hormone levels (THLs) in COPD patients. While some studies have reported normal THLs in stable patients with mild to severe COPD [4,5], others have found low THLs in both regular and hospitalized patients due to COPD exacerbations [6,7]. Thyroid hormone changes are more frequently observed in the Global Initiative for Chronic Obstructive Lung Disease (GOLD) stage 3–4 COPD patients than in the GOLD stage 1–2 [8]. Changes in THLs have been documented in patients admitted to the ICU because of acute hypercapnic respiratory failure (AHRF), and more significant hormonal changes have been observed in intubated patients with COPD than in non-intubated ones [9,10,11]. The extent of thyroid hormone alterations is linked to disease severity, and critically ill COPD patients with high Acute Physiology and Chronic Health Evaluation (APACHE) II scores display marked reductions in THLs [10,12]. The prevalence of changes in THLs ranges from 20% in stable COPD patients to 70% in those with acute respiratory failure [7,9,10,12]. Systemic inflammation is believed to underlie alterations in THLs [13]. Typically, plasma triiodothyronine (T3) levels decrease due to the reduced conversion of thyroxine (T4) to T3, accompanied by normal or low plasma T4 and thyroid-stimulating hormone (TSH) levels, a condition referred to as non-thyroidal illness syndrome (NTIS) [14].

This study aimed to examine thyroid hormone level variations in COPD patients with AHRF admitted to the ICU for mechanical ventilation support and to evaluate the impact of thyroid hormone changes on the clinical outcomes of severely ill COPD patients.

## Materials and Methods

This retrospective study included all patients with COPD admitted to the ICU between March 2015 and April 2018. COPD was diagnosed based on initial pulmonary function tests following the GOLD criteria or through patient history and physical examination findings consistent with chronic hypercapnia and hypoxia at admission [1]. All COPD patients aged ≥ 40 years admitted to the ICU due to AHRF, characterized by pH <7.35 and PCO_2_ >45 mmHg, were included. Patients were excluded based on the following criteria: the presence of hyperthyroidism or hypothyroidism; recent use of medications affecting the hypothalamus-pituitary-thyroid axis before ICU admission, such as corticosteroids, propranolol, potassium iodine, iodinated radiographic contrast agents, amiodarone, lithium, interferon, monoclonal antibodies, and dopamine; other endocrine diseases (except diabetes mellitus [DM]); active cancer; end-stage renal failure; chronic liver disease; recent primary operations before ICU admission; and death or discharge within the first 24 hours after admission. None of the patients with DM experienced diabetic ketoacidosis, and all were diagnosed with type II DM [15]. Ethical approval for this study was obtained from the University Ethics Review Board (no.2018/63). Owing to the study’s retrospective nature, the need for informed consent was waived. The study was carried out in accordance with the Helsinki Declaration.

Patients were admitted from the hospital wards or emergency department. Upon ICU admission, all patients required noninvasive mechanical ventilation (NIMV) and/or invasive mechanical ventilation (IMV) support. Patients with hemodynamic instability, deep coma (Glasgow Coma Scale score [GCS]. <8), life-threatening gas exchange alterations, inability to manage secretions, or NIMV failure were deemed appropriate for IMV. NIMV was administered via a face mask using bilevel-positive airway pressure. All patients underwent chest radiography and/or thoracic computed tomography (CT) before or after ICU admission. Pneumonia was defined as the presence of infiltrates on radiological images. COPD exacerbations (bronchitis) were characterized by the presence of at least two of the following symptoms: dyspnea, coughing, purulent sputum, and increased sputum volume. Urosepsis was diagnosed based on urine culture results, indicating bacterial growth and clinical signs and symptoms of sepsis. Pulmonary embolism was identified using thoracic computed tomography angiography. The primary outcome of this study was to determine changes in circulating THLs. The secondary outcome was to assess the relationship between thyroid hormone variations and morbidity and mortality.

The following parameters were documented: APACHE II score, age, sex, comorbidities, smoking status, cause of respiratory failure, initial laboratory tests (within the first six hours), admission GCS, THLs (TSH, freeT4 [fT4], and free T3 [fT3]), the necessity for IMV or NIMV, renal replacement therapy (RRT) and vasopressor infusion during ICU stay, ICU and hospital length of stay (LOS), and in-hospital mortality. Thyroid hormones were measured within the first 24 h of ICU admission to detect acute changes in the hormone levels without considering a particular time for collecting blood samples. As all patients received oxygen support during their initial day in the ICU, all laboratory measurements, including THLs, were conducted while patients were receiving supplemental oxygen, and the PaO_2_/FiO_2_ ratio was used instead of PaO_2_. Active smoking was defined as smoking at least one cigarette per day until hospital admission. Only the first admission records of patients with multiple ICU admissions were used. Patients were followed-up until death or hospital discharge, whichever occurred first.

Plasma levels of TSH (normal limits:0.3–4.2 μIU/mL), fT4 (normal limits: 0.93–1.7 ng/dL), and fT3 (normal limits:2.0–4.4 pg/mL) were measured using electrochemiluminescence analysis (Elecsys, Roche Diagnostics, Mannheim, Germany) with the Roche Cobas E analyzer. Patients with plasma TSH levels above the upper limit of the normal range and low fT4 levels were considered to have hypothyroidism and were excluded from the study [16]. Patients with plasma TSH levels below 0.3 μIU/mL and elevated plasma fT4 and/or fT3 levels were considered hyperthyroidism and excluded from the study [17]. Seven types of NTIS were diagnosed based on changes in fT3, fT4 and TSH levels: (1) Low fT3, (2) low fT4 (normal fT3 and normal TSH levels), (3) low TSH (normal fT3 and normal fT4 levels), (4) low fT3 and low fT4, (5) low fT3 and low TSH, (6) low TSH and low fT4, and (7) low TSH, low fT3 and low fT4 [6,12]. As thyroid-binding protein concentrations often decrease owing to the acute phase response, fT3 and fT4 were measured in critically ill patients [18].

### Statistical analysis

Descriptive data were presented as means with standard deviations or medians with interquartile ranges. Normally distributed variables were compared using the Student t-test, while the Mann-Whitney U-test was used for variables without a normal distribution. Categorical data, presented as numbers and percentages, were compared using the Pearson chi-squared test and Fisher’s exact test. The relationships between the variables were examined using Pearson’s correlation analysis. Forward logistic regression analysis was performed to identify the independent predictors of in-hospital mortality, and variables yielding p values less than 0.100 at univariate testing were included in the multivariate logistic regression analysis. Odds ratios (OR) were reported with 95% confidence intervals (CI). Statistical significance was determined using a p-value of less than 0.05. Statistical analysis was conducted using Statistical Package for the Social Sciences version 23 (SPSS Inc., Armonk, NY, USA).

## Results

One hundred and one COPD patients with AHRF were admitted to the ICU during the study period. Twenty-one patients were excluded because of recent major surgery (n=6), hypothyroidism (n=5), hyperthyroidism (n=5), and corticosteroid use (n=5). Eighty patients were included in the study, all of whom underwent plasma TSH, fT4, and fT3 measurements. The average age of the patients was 71.5 ± 9.5 years, and 57.5% were male. Forty-seven patients (64%) were smokers, with a mean 70.4 ± 45.7 pack-year, and 18 (38.3%) were currently smoking. The cause of COPD in the remaining patients was exposure to indoor and outdoor air pollution. Half of the patients had received long-term oxygen therapy. The patients’ average APACHE II score and admission GCS were 22.8 ± 7.4 and 11.5 ± 3.9, respectively. The causes of AHRF were bronchitis (45%), pneumonia (33.7%), hearth failure (10%), noncompliance with treatment (7.5%), urosepsis (2.5%), and pulmonary embolism (1.3%). All patients with urosepsis (n=2) were women and had type II DM. ICU and hospital LOS were 6 (4–6) and 11 (6–16) days, respectively. The patients’ average TSH, fT4, and fT3 levels were 1.00 ± 0.99 μIU/mL, 1.07 ± 0.32 ng/dL, and 2.29 ± 0.77 pg/mL, respectively.

In 51 patients (63.7%), NTIS was diagnosed, with low plasma fT3 levels being mainly encountered (Table 1). Patients with NTIS had lower serum albumin levels, higher white blood cell counts (WBC), and an increased in-hospital mortality rate than those without NTIS (Table 2). Low plasma fT3 levels were observed in 29 (36.3%) patients, low fT4 in 27 (33.8%), and low TSH in 12 (15%) patients, respectively. Patients with low plasma fT3 levels had elevated C-reactive protein (CRP) levels, and WBC (Table 2). Moreover, patients with low plasma fT3 levels had high APACHE II scores, were predominantly admitted from the wards, required more vasopressor infusion during their ICU stay, spent a longer period of time in the ICU, and had a high in-hospital mortality rate. Patients with low plasma TSH levels had a lower GCS score than those with normal plasma TSH levels, and there were no differences between patients with and without low plasma fT4 levels (Table 3). No relationship was observed between low THLs and the causes of AHRF. Seven patients received RRT in the ICU, and statistical analysis revealed no differences between NTIS and RRT requirements during ICU stay. The THLs were similar between active smokers and non-smokers.

**Table 1.** Thyroid hormone results of COPD patients with acute hypercapnic respiratory failure

**Parameters**	**N (%)**
Normal thyroid hormone levels	29 (36.3)
Non-thyroidal illness syndrome	
Low fT3	17 (21.2)
Low fT4	14 (17.5)
Low TSH	4 (5)
Low fT3 and fT4	8 (10)
Low TSH and fT3	3 (3.7)
Low TSH and fT4	4 (5)
Low TSH, fT3 and fT4	1 (1.3)
Total	80 (100%)

**Abbreviations:** COPD, chronic obstructive pulmonary disease; fT3, free triiodothyronine; fT4, free thyroxine; N, number; TSH, thyroid-stimulating hormone.

**Table 2.** A comparison of acute hypercapnic respiratory failure COPD patients with and without non-thyroidal illness syndrome

**Parameters**	**NTIS (−) (N = 29)**	**NTIS (+) (N = 51)**	**p**
Age, years	70.5 ± 7.3	71.2 ± 9.5	0.445
Male, n (%)	17 (58.6)	29 (56.9)	0.878
HT, n (%)	18 (62.1)	32 (62.7)	0.952
DM, n (%)	11 (37.9)	12 (23.5)	0.171
CVD, n (%)^†^	24 (82,8)	35 (68.6)	0.167
Long-term O_2_ use, n (%)	14 (48.3)	26 (51.0)	0.816
Admission from wards, n (%)^‡^	12 (41.4)	30 (58.8)	0.133
APACHE II	21.7 ± 7.0	23.4 ± 7.7	0.295
GCS	11.6 ± 3.8	11.4 ± 4.1	0.829
IMV, n (%)	13 (44.8)	22 (43.1)	0.884
Vasopressor, n (%)	8 (27.6)	20 (39.2)	0.294
Albumin, g/dL	3.5 ± 0.5	3.1 ± 0.5	**0.011**
Creatinine, mg/dL	1.5 ± 1.3	1.3 ± 1.4	0.635
CRP, mg/dL	6.2 (1.6–12)	9.7 (1.9–14.8)	0.519
WBC, ×10^3^/L	11.1 ± 3.2	13.4 ± 7.0	**0.044**
pH	7.28 ± 0.06	7.26 ± 0.09	0.392
PCO_2_, mm Hg	64.0 ± 17.1	66.2 ± 17.0	0.545
HCO_3_, mEq/L	25.5 ± 4.6	25.7 ± 6.1	0.886
PaO_2_/FiO_2_, mm Hg	227 ± 97	202 ± 88	0.251
ICU LOS, days	6 (4–10.5)	5 (4–10)	0.693
Hospital LOS, days	12 (7.5–14.5)	10 (6–16)	0.634
In-hospital mortality, n (%)	4 (13.8)	19 (37.3)	**0.022**

† Including heart failure, ischemic heart disease, any arrhythmia, and heart valve diseases.

‡ The remaining patients were admitted to the ICU from the emergency department.

Abbreviations: APACHE, Acute Physiology and Chronic Health Evaluation; CVD; cardiovascular disease; CRP, C-reactive protein; DM, diabetes mellitus; GCS, Glasgow coma scale; HT, hypertension; IMV, invasive mechanical ventilation; LOS, length of stay; N, number; NTIS, non-thyroidal illness; WBC, white blood cell count.

**Table 3.** A comparison of acute hypercapnic respiratory failure COPD patients with and without low thyroid hormone levels

**Parameters**	**TSH**			**fT4**			**fT3**		
	**≥0.3 IU/mL (N = 68)**	**<0.3 IU/mL (N = 12)**	**P**	**≥0.93 ng/dL (N = 53)**	**<0.93 ng/dL (N = 27)**	**P**	**≥2 pg/mL (N = 51)**	**<2 pg/mL (N = 29)**	**P**
Age, years	70.9 ± 9.6	75.3 ± 8.3	0.119	71.9 ± 8.9	70.7 ± 10.7	0.618	71.5 ± 7.0	71.7 ± 10.3	0.936
Male, n (%)	39 (57.4)	7 (58.3)	0.949	28 (52.8)	18 (66.7)	0.236	31 (60.8)	5 (51.7)	0.431
HT, n (%)	42 (61.8)	8 (66.7)	0.746	35 (66.0)	15 (55.6)	0.360	30 (58.8)	20 (69)	0.368
DM, n (%)	21 (30.9)	2 (16.7)	0.493	18 (34.0)	5 (18.5)	0.195	17 (33.3)	6 (20.7)	0.230
CVD, n (%)^†^	39 (57.4)	6 (50.0)	0.755	33 (62.3)	12 (44.4)	0.156	31 (60.8)	14 (48.3)	0.278
Long-term O_2_ use, n (%)	31 (45.6)	9 (75.0)	0.060	28 (52.8)	12 (44.4)	0.637	25 (49.0)	15 (51.7)	0.816
Admission from wards, n (%)^‡^	35 (51.5)	7 (58.3)	0.611	26 (49.1)	16 (59.3)	0.480	22 (43.1)	20 (69.0)	**0.026**
APACHE II	22.7 ± 7.5	23.3 ± 7.3	0.816	23.0 ± 7.2	23.4 ± 8.0	0.632	20.9 ± 7.0	26.1 ± 7.1	**0.002**
GCS	11.9 ± 3.7	9.0 ± 4.2	**0.042**	11.6 ± 3.7	11.1 ± 4.3	0.570	11.9 ± 3.7	10.7 ± 4.1	0.184
IMV, n (%)	30 (44.1)	5 (41.7)	0.875	22 (41.5)	13 (48.1)	0.710	19 (37,3)	16 (55.2)	0.120
Vasopressor, n (%)	25 (36.3)	3 (25)	0.912	19 (35.9)	9 (33.3)	0.823	12 (23.5)	16 (55.2)	**0.004**
Albumin, g/dL	3.3 ± 0.5	3.2 ± 0.6	0.590	3.3 ± 0.5	3.1 ± 0.6	0.209	3.4 ± 0.5	3.0±0.5	0.059
Creatinine, mg/dL	1.4 ± 1.4	1.3 ± 0.7	0.691	1.4 ± 1.1	1.4 ± 1.4	0.660	1.3 ± 1.1	1.5 ± 1.4	0.473
CRP, mg/dL	7.6 (2–13)	7.9 (1.5–18)	0.740	8 (2–17)	10 (1–13)	0.791	5.5 (1–12)	11 (4.6–16)	**0.010**
WBC ×10^3^/L	14 ± 7.5	13.3 ± 4.8	0.668	13.8 ± 6.7	14.1 ± 8.1	0.887	12.6 ± 5.0	16.2 ± 9.5	**0.027**
pH	7.26 ± 0.08	7.29 ± 0.07	0.108	7.28 ± 0.05	7.25 ± 0.10	0.085	7.28 ± 0.07	7.25 ± 0.08	0.102
PCO_2_, mm Hg	66 ± 17.4	62.8 ± 14.9	0.514	65.5 ± 16.4	65.6 ± 18.4	0.996	64 ± 18	68.0 ± 15.0	0.360
HCO_3_, mEq/L	25.3 ± 5.5	27.1 ± 6.0	0.376	26.2 ± 4.8	24.4 ± 6.7	0.178	25.6 ± 5.0	25.5 ± 6.6	0.920
PaO_2_/FiO_2_, mm Hg	215±92	192 ± 93	0.448	205 ± 92	223 ± 93	0.429	224 ± 95	190 ± 84	0.100
ICU LOS, days	6 (4–10.3)	5 (4–9)	0.554	6 (4–10.5)	5 (4–9)	0.561	5 (4–7)	8 (4–20)	**0.021**
Hospital LOS, days	10 (6–15.3)	11 (6–20.5)	0.765	11 (7.5–15.5)	10 (6–16)	0.415	9 (6–14)	11 (9–23)	0.142
In-hospital mortality, n (%)	19 (27.9)	4 (33.3)	0.206	16 (30.2)	7 (25.9)	0.797	9 (17.6)	14 (48.3)	**0.004**

† Including heart failure, ischemic heart disease, any arrhythmia, and heart valve diseases.

‡ The remaining patients were admitted to the ICU from the emergency department.

***Abbreviations:*** APACHE, Acute Physiology and Chronic Health Evaluation; CVD, cardiovascular disease; CRP, C-reactive protein; DM, diabetes mellitus; fT3, free triiodothyronine; fT4, free thyroxine; GCS, Glasgow coma scale; HT, hypertension; IMV, invasive mechanical ventilation; LOS, length of stay; N, number; TSH, thyroid-stimulating hormone; WBC, white blood cell count.

The in-hospital mortality rate was 28.8%. Non-survivors had low fT3 (1.75 ± 0.60 vs 2.50 ± 0.73 pg/mL; p <0.001) and albumin (2.9 ± 0.6 vs 3.4 ± 0.5 g/dL; p <0.001) levels, but high creatinine (1.9 ± 2.1 vs 1.2 ± 0.8 mg/dL; p=0.028) and CRP (10.8 (5.4–16.2) vs 6.2 (1.4–12.6) mg/dL; p=0.022) levels, along with high APACHE II scores (28.6 ± 5.7 vs 20.4 ± 6.7; p <0.001) and low GCS (10.0 ± 3.4 vs 12.0 ± 4.0; p=0.032). They also required IMV (73.9% vs 31.6%, p=0.001), RRT (26.1% vs 1.8%, p=0.002), and vasopressors (73.9% vs 19.3%, p <0.001) more frequently during their ICU stay. Multivariate logistic regression analysis identified fT3, APACHE II and vasopressor use as prognostic indicators of in-hospital mortality (Table 4). Correlation analysis revealed significant negative associations between fT3 and APACHE II scores (r=−0.377, p=0.001), CRP (r=−0.361, p=0.001), and albumin (r=0.406, p <0.001), as well as a positive relationship between fT4 and pH (r=0.299, p=0.007).

**Table 4.** Univariate and multivariate logistic regression analyses for estimating in-hospital mortality

**Parameters**	**Univariate^†^**			**Multivariate^‡^**		
	**P**	**OR**	**95% CI**	**P**	**OR**	**95% CI**
fT3	0.001	0.172	0.062–0.473	0.027	0.271	0.085–0.865
IMV	0.001	6.139	2.073–18.175			
Albumin	<0.001	0.094	0.026–0.344			
Creatinine	0.063	1.490	0.979–2.270			
CRP	0.041	1.048	1.002–1.097			
WBC	0.044	1.072	1.002–1.147			
APACHE II	<0.001	1.210	1.099–1.334	0.007	1.155	1.041–1.282
GCS	0.043	0.880	0.777–0.996			
Vasopressor	<0.001	11.848	3.790–37.037	0.013	5.426	0.439–20.468
Admission from the ward	0.056	2.725	0.973–7.632			

† Those variables with p values of less than 0.100 when tested in univariate analysis are listed in the table.

‡ Hosmer-Lemeshow: X^2^, 2.937; df, 8; p=0.938.

***Abbreviations:*** APACHE, Acute Physiology and Chronic Health Evaluation; CI, confidence interval; CRP, C-reactive protein; fT3, free triiodothyronine; fT4, free thyroxine; GCS, Glasgow coma scale; IMV, invasive mechanical ventilation; OR, odds ratio; TSH, thyroid-stimulating hormone; WBC, white blood cell count.

## Discussion

Acute medical conditions often trigger fluctuations in circulating THLs, with a decline in plasma T3 levels being the most frequently observed change. Additionally, in severe and prolonged disease states, plasma T4 and TSH levels may decrease [18,19]. Numerous acute illnesses, such as cardiovascular, cerebrovascular, gastrointestinal, and infectious diseases; surgery; trauma; cancer; and burns, can lead to alterations in THLs [14,20]. The extent of these changes, particularly reductions in T3 levels, generally reflects the severity of the disease and correlates with mortality [21]. Mediators released during acute disease states, including interleukin-6, tumor necrosis factor-α, and interleukin-1, have been implicated in alterations in circulating THLs [13, 14].

The current study assessed critically ill COPD patients with AHRF requiring mechanical ventilation, and NTIS was diagnosed in 63.7% of cases. Patients with low plasma fT3 levels exhibited higher APACHE II scores and elevated inflammation markers (CRP and WBC), required more extensive organ support therapy, and had a higher mortality rate than patients with low plasma TSH and fT4 levels. Plasma fT3 levels were also identified as a predictor of mortality. Our findings are consistent with previous studies investigating THLs in critically ill patients. A study by Wang et al., which included 480 unselected ICU patients, identified fT3 levels as an independent predictor of ICU mortality and demonstrated a negative correlation between CRP and fT3 levels [22]. Scioscia et al. examined 32 patients with severe respiratory failure and reported lower plasma fT3 levels in non-survivors than in survivors [23]. These authors also found a negative correlation between the APACHE II scores and plasma fT3 levels. A prospective study by Rothberger et al., which involved 162 patients requiring IMV due to various causes, showed low plasma fT3 levels in 60% of patients and reported a significantly higher mortality rate among patients with low plasma fT3 levels [24].

Furthermore, the current study’s outcomes align with earlier research on COPD patients admitted to the hospital or ICU due to acute respiratory failure. Bacakoğlu et al. reported a 43% rate of NTIS in patients with exacerbated COPD. [9]. In addition, they found a notably higher rate of IMV in patients with thyroid hormone dysregulation than in those without such impairments. In a study by Yaşar et al., which included 127 intubated COPD patients, NTIS was identified as a predictor of prolonged intubation, and higher APACHE II scores were observed in patients with NTIS [12]. Karadağ et al.’s prospective study reported a 70% rate of NTIS in exacerbated COPD patients compared to 20% in stable COPD patients, with significant reductions in plasma T3 and TSH levels in exacerbated COPD patients [7]. Madhuri et al. compared the THLs of stable and exacerbated COPD patients to those of healthy subjects and found that patients with COPD exacerbation had significantly reduced fT3 and TSH levels, while stable COPD patients had low mean fT3 levels, but still within normal limits [25]. Ergan et al.’s study on severely ill elderly patients (≥65 years) with acute respiratory failure due to COPD exacerbation reported low plasma T3 levels in 65.9% of patients, low plasma T4 levels in 9%, and a negative correlation between plasma T3 levels and APACHE II scores [10]. These authors also observed lower plasma T3 levels in patients who underwent IMV than in those who received NIMV, and non-survivors had lower plasma T3 levels than survivors. Khalil et al.’s study of 48 COPD patients admitted to the ICU due to acute exacerbation revealed thyroid dysfunction in 25% [26]. All patients with thyroid dysfunction exhibited decreased fT3 levels with normal plasma fT4 and TSH levels, along with high APACHE II scores. In addition, the mortality risk increased 2.1-fold in the presence of thyroid dysfunction. Gümüş et al. examined 132 patients with COPD hospitalized for acute exacerbation and identified NTIS in 73 (55%) patients [6]. Among the 73 patients, 40 had low TSH levels, 24 had low T3 levels, four had low T4 levels, three had both low T3 and T4 levels, and two had low T3, T4, and TSH levels.

### Limitations

This study had several limitations that should be acknowledged. First, this was a retrospective investigation with a relatively small patient population. Second, data on FEV1 and the FEV1/FVC ratio, representing the severity of airflow obstruction in COPD patients and significantly influencing mortality, were unavailable. Third, information regarding extubation failure, a crucial prognostic indicator of mortality in critically ill intubated patients, was also absent. Fourth, all laboratory parameters were examined while the patients were receiving supplemental oxygen, making it impossible to establish any connection between baseline hypoxia and THLs. Lastly, since glycosylated HbA1c data for patients with diabetes were not available, we could not investigate the relationship between serum THLs and glycosylated HbA1c.

## Conclusion

Significant alterations in circulating THLs were observed in critically ill COPD patients with AHRF. While changes were evident in all three hormone levels, only reductions in plasma fT3 levels were associated with disease severity and mortality. Therefore, plasma fT3 levels should be considered a reliable indicator of disease severity in patients with COPD who have acute respiratory failure.
